# Association between accompanying duration and anxiety/depression among family caregivers: a prospective cohort study in China during the COVID-19 pandemic

**DOI:** 10.3389/fpsyt.2024.1411500

**Published:** 2024-11-08

**Authors:** Yanhong Jiang, Weiwen Hong, Lili Huang, Hongan Ying, Weiwei Hu

**Affiliations:** ^1^ Department of Outpatient, Taizhou First People's Hospital, Taizhou, Zhejiang, China; ^2^ Department of General Surgery, Huangyan Traditional Chinese Medicine Hospital, Taizhou, Zhejiang, China; ^3^ Department of Emergency, Taizhou First People's Hospital, Taizhou, Zhejiang, China; ^4^ Department of Cardiovascular Medicine, Huangyan Traditional Chinese Medicine Hospital, Taizhou, Zhejiang, China; ^5^ Department of General Surgery, Taizhou First People's Hospital, Taizhou, Zhejiang, China

**Keywords:** accompanying days, anxiety, depression, family caregivers, prospective cohort

## Abstract

**Introduction:**

While some studies have explored family caregivers’ anxiety and depression, limited research has been conducted on family caregivers’ anxiety and depression caused by the duration of companionship, resulting in an unclear relationship between the length of accompaniment and the psychological well-being of family caregivers.

**Methods:**

This cohort study was conducted from April 1, 2022, to June 30, 2022 in general surgery. We employed bar graphs and line graphs to illustrate the relationship between accompanying days and anxiety and depression. Additionally, mixed-effects linear regression models were utilized to examine the correlation between accompanying days and the likelihood of anxiety and depression.

**Results:**

The study had 207 family caregivers, with 23.5% experiencing anxiety and 13.1% experiencing depression. Anxiety and depression scores peaked on day 9, and the incidence rate was the highest for the third group (≥10 days). Family caregivers in the first group (≤4 days) of companionship had significantly higher anxiety (β=0.27, 95% CI 0.16-0.39, *p*<0.001 in all three models). Those in the second group (5-9 days) and the third group (≥10 days) showed no significant correlation with anxiety scores, except for a negative correlation in Model III (β=-0.15, 95% CI -0.29 to -0.01) for companionship in the third group (≥10 days). Family caregivers in the first group (≤4 days) of companionship had significantly higher depression scores (β=0.19, 95% CI 0.10-0.29, *p*<0.001 in all three models). Those in the second group (5-9 days) had no significant relationship with depression, while those in the third group (≥10 days) exhibited a small negative correlation in Model II and III (β= -0.02, 95% CI -0.08-0.04). The sensitivity analysis confirms the mixed-effects linear regression findings.

**Conclusion:**

There was a positive correlation between the duration of family companionship and anxiety and depression in the early days.

## Introduction

Family caregiving refers to the provision of unpaid assistance and support to a family member or loved one who has a chronic illness, disability, or other health condition (1, 2). While providing care for a family member can be rewarding, it can also be a source of stress and strain (3–5). While research has shown that family caregivers are at an increased risk of developing anxiety and depression due to the stress of caring for a loved one (6), studies specifically investigating how caregiver anxiety and depression vary with the duration of hospital accompaniment remain scarce.

Anxiety and depression are among the most common mental health conditions, affecting millions of people worldwide. The harms associated with anxiety and depression can include physical health problems, social isolation, and decreased quality of life. In severe cases, anxiety and depression can lead to suicidal thoughts and behaviors. Research has consistently shown that family caregivers are at a higher risk of experiencing symptoms of anxiety and depression compared to non-caregivers (7–9). A meta-analysis of 84 studies found that caregivers had significantly higher levels of depression and anxiety compared to non-caregivers (10). The psychological status of family caregivers can have a significant impact on the patient’s prognosis and overall health outcomes. Family caregivers’ stress has been linked to increased risk of hospitalization, as well as increased mortality rates for the patient (11). Family caregivers’ stress can affect the quality of care that the patient receives. Family caregivers who are experiencing high levels of stress or burnout may be less able to provide effective care, which could lead to medication errors, missed appointments, or other lapses in care that could negatively impact the patient’s health outcomes (12).

The prolonged accompanying time required during the COVID-19 pandemic may contribute to increased anxiety among patients’ family members (13). With restrictions on social gatherings and limited visitation policies in hospitals and clinics, family caregivers may be required to spend more time with their loved ones than they would under normal circumstances. This can be especially challenging for family caregivers who are simultaneously juggling work and other responsibilities (14). The pandemic has highlighted the need for increased support and resources for family caregivers to help them cope with the additional stresses and mental health challenges they face during these unprecedented times.

Understanding the relationship between the duration of family companionship and the prevalence of anxiety and depression is essential for developing effective support and interventions for family caregivers. Recognizing the impact of family companionship duration on anxiety and depression can reduce the occurrence of these conditions, improve the quality of care for family companions, and provide a clinical basis for adjusting the timing of family companionship.

## Methods

### Data collection

This study gathered data from family members who accompanied patients admitted to the Department of General Surgery at the First People’s Hospital in Taizhou, Zhejiang, China. from April 1, 2022, to June 20, 2022. The study was conducted in the Department of General Surgery at the First People’s Hospital of Taizhou, a tertiary-level A hospital affiliated with Wenzhou Medical University in Zhejiang Province, China. In the Chinese hospital classification system, tertiary-level A (also known as Grade 3A) hospitals are the highest-ranked medical institutions, offering comprehensive, specialized medical services and serving as major referral centers. Tertiary-level A hospitals in China are comparable to university hospitals or large medical centers in other countries. The Department of General Surgery primarily performs surgeries for acute abdominal conditions, varicose veins, and hernias. The annual inpatient volume of the department is approximately 2,000. In addition to collecting data on patient demographics, this study also gathered information on potential risk factors for anxiety and depression symptoms, such as gender, age, education, religious affiliation, residential and occupational history, smoking and alcohol habits, hypertension, and diabetes from the family caregivers. Three trained and specialized data collectors utilized a structured questionnaire to carry out data collection. On the first day of admission, demographic data and information on potential risk factors for anxiety symptoms were gathered. Subsequently, the Hospital Anxiety and Depression Scale (HADS) was administered to family caregivers once daily until this patient discharge or transfer to collect anxiety and depression data. This study was registered with the registration number MR-33-24-026591 and our study was conducted with full ethical approval from the Medical Ethics Committee of Taizhou First People’s Hospital (approval number: 2022-KY004-02).

### Participants

**Figure 1:**
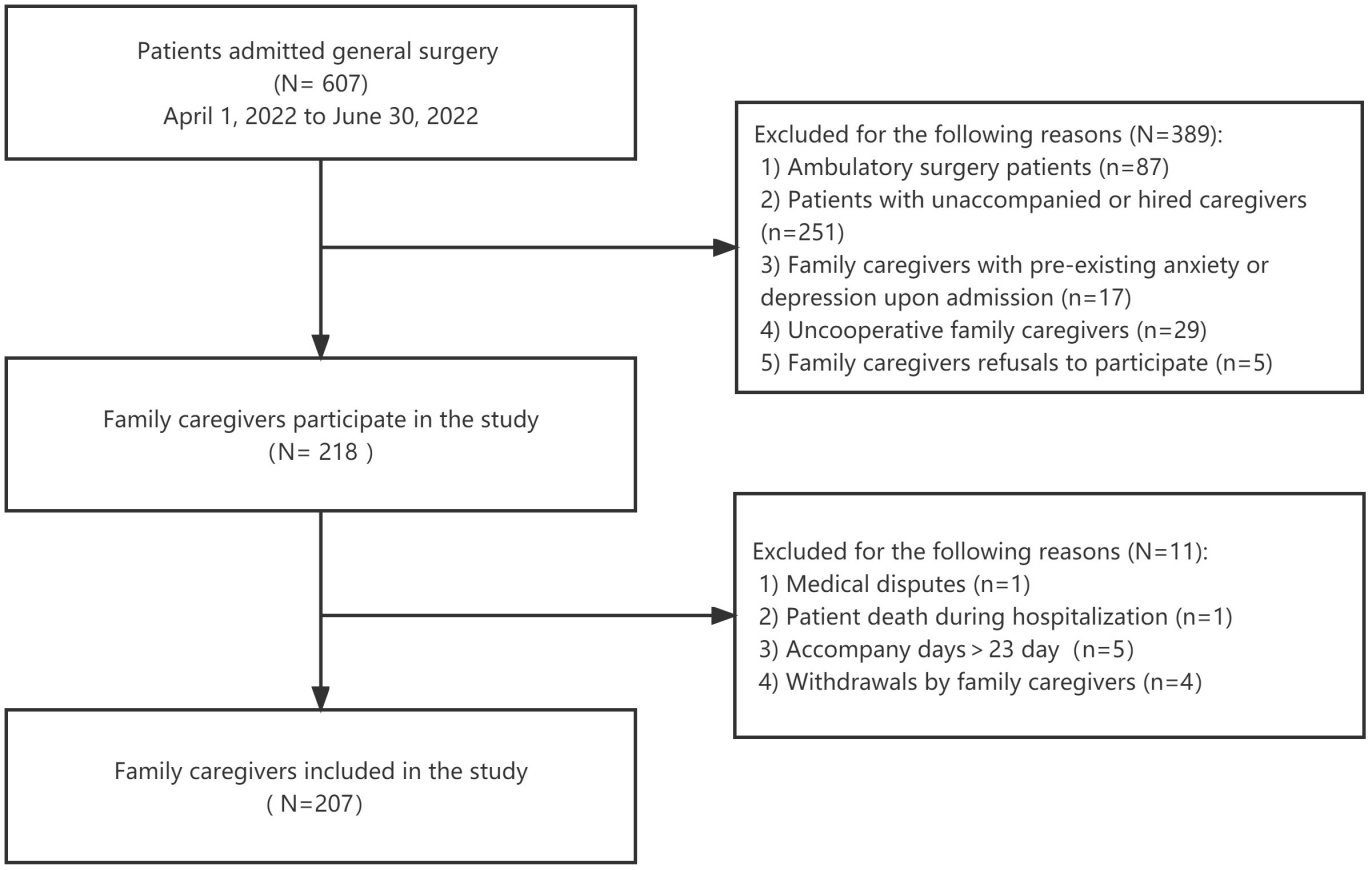
The flow chart of the study.

Out of 607 patients admitted to the general surgery department between April 1, 2022 and June 30, 2022, 87 were day patients, 251 were unaccompanied or accompanied by someone other than a family member, 17 family caregivers had pre-existing anxiety or depression assessed by HADS upon admission, 29 family caregivers were unable to complete the questionnaire due to various reasons, and 5 family caregivers refused to participate in the study. The main disease categories of the patients included acute appendicitis, acute pancreatitis, intestinal obstruction, gastrointestinal perforation, incarcerated hernia, and peripheral vascular diseases such as varicose veins and lower extremity arterial occlusive disease. Consequently, 218 patients and their family caregivers provided informed consent to participate in the study. Following an average follow-up period of 5.5 days, there was one in-hospital death, one medical dispute, five cases with a length of stay exceeding 23 days, and four cases where informed consent was withdrawn. Finally, the study ultimately included 207 family caregivers (**Figure 1**).

### Definition of anxiety and depression

Anxiety and depression were defined using the Hospital Anxiety and Depression Scale (HADS) in this study (15). The Hospital Anxiety and Depression Scale (HADS) is a widely used self-report questionnaire designed to assess symptoms of anxiety and depression in individuals receiving medical care (16). The HADS comprises 14 items, with seven items each for anxiety and depression. Each item is rated on a 4-point scale ranging from 0 to 3, with higher scores indicating greater severity of symptoms. The anxiety subscale of the HADS evaluates symptoms such as feelings of tension or nervousness, worry, and fearfulness. Sample items from the anxiety subscale include “I feel tense or ‘wound up’” and “I get a sort of frightened feeling as if something awful is about to happen (17)”.

The depression subscale of the HADS evaluates symptoms such as low mood, anhedonia, and feelings of worthlessness or hopelessness. Sample items from the depression subscale include “I have lost interest in my appearance” and “I feel as if I am slowed down.” The HADS was specifically developed for use in medical settings and has demonstrated good reliability and validity in measuring symptoms of anxiety and depression among patients with physical illness. As a widely used screening tool, the HADS can facilitate the identification of individuals who may require further assessment or treatment for mental health issues (18).

The HADS generates total scores ranging from 0 to 42, with higher scores indicating greater severity of symptoms. A score of 0-7 on either subscale is considered normal, 8-10 is considered borderline abnormal, and 11 or higher is considered abnormal (19). In our study, we included both borderline and abnormal scores (scores ≥8) as positive cases for anxiety and depression (20, 21).

It is important to note that the HADS was not designed to provide a definitive diagnosis of anxiety or depression but rather to serve as a screening tool for identifying individuals who may require further evaluation by healthcare professionals due to the presence of symptoms (22).

### Assessment of pre-existing anxiety and depression of family caregivers

To assess pre-existing anxiety and depression, we employed a two-step approach. First, family caregivers completed a self-reported questionnaire during the baseline survey, indicating if they had ever been diagnosed with anxiety or depression and if they were currently receiving treatment for these conditions. Second, family caregivers who scored ≥8 on either the anxiety or depression subscale of the HADS at baseline underwent a clinical interview with a mental health professional to determine if they had pre-existing anxiety or depression that had not been previously diagnosed or reported. Family caregivers were excluded from the study if they self-reported a previous diagnosis of anxiety or depression in the baseline survey or if pre-existing anxiety or depression was confirmed through the clinical interview process.

### Definition of accompanying days

A family caregiver is defined as a person who provides unpaid care and support to a family member or friend who has a physical, mental or emotional disability, chronic illness or needs related to aging. Family caregivers can take on a variety of roles and responsibilities, such as providing personal care (e.g., bathing, dressing, feeding), instrumental support (e.g., transportation, housework), medication management, and emotional support (23). In this study, one day of accompanying care was defined as exceeding 8 hours of daily accompanying time, with the day of admission being counted as one day regardless of its duration (24, 25), and the day of discharge not being included in the calculation.

### Covariates

This study will measure several covariates for both family caregivers and patients. The covariates for family caregivers encompass a range of demographic, health-related, and social factors, including gender, relationship with the patient, marital status, nationality, occupation, economic difficulties, educational level, height, weight, smoking status, alcohol consumption habits, hypertension status, diabetes status, and coronary heart disease status. Additionally, the study will consider whether the caregiver is accompanying for the first time or not and whether they have children with care needs at home or not. Other factors that will be assessed including whether the caregiver has experienced family conflicts related to the patient’s care and whether they live with the patient or have siblings who share caregiving responsibilities (26, 27). Finally, religious beliefs will also be taken into account as a potential covariate in this study.

As a covariate for patients, this study will measure gender, occupation type, and education level attained. In addition to these factors, the study will also record the medical insurance type and disease type as potential covariates. Marital status was divided into four categories: married, never married, living with a partner, and other (including widowed, divorced, or separated). Diabetes was classified as the use of anti-diabetic medication or a fasting glucose level of 126 mg/dL (7.0 mmol/L) or higher. Smoking status was divided into two groups: never smoked (or smoked less than 100 cigarettes in their whole life) and smoker (smoked at least 100 cigarettes in their whole life). Alcohol drinking status was assessed through the question, “In any year, have you had at least 12 drinks of any type of alcoholic beverage in your lifetime?” Those who responded positively were identified as alcohol drinkers. Coronary artery disease (CAD) was defined as the presence of ischemic heart disease, acute myocardial infarction, or other specified forms of CAD, such as coronary atherosclerosis and chronic total occlusion of coronary arteries (28, 29).

### Statistical analysis

Descriptive statistics will be used to summarize family caregivers characteristics and mental health outcomes. Continuous variables will be presented as means ± standard deviations or medians with interquartile ranges depending on their distribution. Categorical variables will be presented as frequencies and percentages. In order to investigate differences, line graphs were employed to illustrate changes in mean daily anxiety and depression scores while bar graphs were used to display alterations in the percentage of family companionship anxiety and depression with variations in days of companionship. We utilized linear mixed-effect models to analyze the data. We employed three different models to assess the effect of family accompaniment on anxiety and depression: Model I for unadjusted variable model, Model II adjusted for age and gender, and Model III adjusted for all variables. Additionally, since each family caregivers’ accompaniment days differed, we divided family caregivers into three groups according to their accompanying days: first group (≤4 days), second group (5-9 days) and third group (≥10 days). All statistical analyses were performed with R, version 4.0.5(R Project for Statistical Computing) using the survey package, version 4.1-1 and with Free Software Foundation statistics software, version 1.3. In all tests, *p* < 0.05 (2-sided) was considered to indicate statistical significance.

## Results

### Characteristics of the family caregivers

**Table 1** presents the baseline characteristics of 207 family caregivers, divided into three groups based on accompanying days: the first group (≤4 days) (74 family caregivers), the second group (5-9 days) (91 family caregivers), and the third group (≥10 days) (42 family caregivers). While some differences were observed in the proportion of female family caregivers, unemployment rates, education levels, and first-time caregiver status, most of these differences were not statistically significant. The median age, BMI, ethnicity, marital status, lifestyle factors, economic status, and caregiver-patient relationships were similar across the groups. The only significant difference was the higher proportion of first-time family caregivers in the third group (≥10 days) and the first group (≤4 days) compared to the second group (5-9 days) (*p*=0.03).

**Table 1:** Baseline characteristics of family caregivers stratified by accompanying days.

Variables	Total (n = 207)	Accompanying days			*P-value for t-test or χ2-test*
		≤4 (n = 74)	5~9 (n = 91)	≥10 (n = 42)	
**Age, Mean ± SD**	55.2 ± 13.7	54.6 ± 13.9	55.8 ± 14.2	55.0 ± 12.6	0.869
**Sex, n (%)**					0.607
Male	71 (34.3)	25 (33.8)	34 (37.4)	12 (28.6)	
Female	136 (65.7)	49 (66.2)	57 (62.6)	30 (71.4)	
**BMI, Mean ± SD**	23.7 ± 3.5	24.3 ± 4.0	23.2 ± 3.3	23.5 ± 2.9	0.171
**Work, n (%)**					0.408
Employed	91 (44.0)	31 (41.9)	40 (44)	20 (47.6)	
Unemployed	70 (33.8)	26 (35.1)	27 (29.7)	17 (40.5)	
Other	46 (22.2)	17 (23)	24 (26.4)	5 (11.9)	
**Race, n (%)**					1
Han	205 (99.0)	73 (98.6)	90 (98.9)	42 (100)	
Other	2 (1.0)	1 (1.4)	1 (1.1)	0 (0)	
**Education, n (%)**					0.528
Primary school and below	98 (47.3)	32 (43.2)	47 (51.6)	19 (45.2)	
Secondary school	88 (42.5)	31 (41.9)	37 (40.7)	20 (47.6)	
College or above	21 (10.1)	11 (14.9)	7 (7.7)	3 (7.1)	
**Maritalstatus, n (%)**					0.65
Single	16 (7.7)	4 (5.4)	8 (8.8)	4 (9.5)	
Married	191 (92.3)	70 (94.6)	83 (91.2)	38 (90.5)	
**Smoke, n (%)**	43 (20.8)	14 (18.9)	22 (24.2)	7 (16.7)	0.542
**Drink, n (%)**	34 (16.4)	12 (16.2)	16 (17.6)	6 (14.3)	0.891
**Religion, n (%)**	117 (56.5)	38 (51.4)	55 (60.4)	24 (57.1)	0.502
**Economy, n (%)**					0.42
No economic difficulties	180 (87.0)	66 (89.2)	76 (83.5)	38 (90.5)	
Economic difficulties	27 (13.0)	8 (10.8)	15 (16.5)	4 (9.5)	
**Hypertension, n (%)**	38 (18.4)	16 (21.6)	12 (13.2)	10 (23.8)	0.225
**Diabetes, n (%)**	14 (6.8)	8 (10.8)	4 (4.4)	2 (4.8)	0.240
**Hyperlipidemia, n (%)**	12 (5.8)	7 (9.5)	4 (4.4)	1 (2.4)	0.272
**Stroke, n (%)**	4 (1.9)	2 (2.7)	1 (1.1)	1 (2.4)	0.667
**CVD, n (%)**	5 (2.4)	3 (4.1)	1 (1.1)	1 (2.4)	0.435
**Cancer, n (%)**	9 (4.3)	3 (4.1)	4 (4.4)	2 (4.8)	1
**First nursing, n (%)**	80 (38.6)	35 (47.3)	26 (28.6)	19 (45.2)	0.030
**Relation, n (%)**					0.392
Spouse	112 (54.1)	42 (56.8)	50 (54.9)	20 (47.6)	
Daughter/son	61 (29.5)	22 (29.7)	22 (24.2)	17 (40.5)	
Parents	19 (9.2)	4 (5.4)	12 (13.2)	3 (7.1)	
Brothers/Sisters	4 (1.9)	3 (4.1)	1 (1.1)	0 (0)	
Other	11 (5.3)	3 (4.1)	6 (6.6)	2 (4.8)	
**Only child, n (%)**	33 (15.9)	13 (17.6)	11 (12.1)	9 (21.4)	0.350
**family conflict, n (%)**	6 (2.9)	3 (4.1)	3 (3.3)	0 (0)	0.603
**Childcare, n (%)**	67 (32.4)	30 (40.5)	25 (27.5)	12 (28.6)	0.171
**Living with patients, n (%)**	153 (73.9)	55 (74.3)	68 (74.7)	30 (71.4)	0.918
**Anxious, Mean ± SD**	3.4 ± 3.2	2.2 ± 2.5	2.8 ± 2.9	4.6 ± 3.3	< 0.001
**Depression, Mean ± SD**	2.6 ± 2.8	1.7 ± 2.3	2.1 ± 2.6	3.5 ± 2.9	< 0.001
**Anxious (%)**					< 0.001
No	76.5	94.5	81.9	63.7	
Yes	23.5	5.5	18.1	36.3	
**Depression (%)**					< 0.001
No	86.9	90.1	89.4	83.1	
Yes	13.1	9.9	10.6	16.9	

Means and standard error were described for the continuous variables, counts and proportions were described for categorical variables.

In the entire cohort, the occurrence of anxiety was 23.5%, accompanied by a mean HADS score (± SD) of 3.4 (± 3.2). The incidence of depression in all family caregivers was 13.1%, with a mean HADS score (± SD) of 2.6 (± 2.8). The prevalence of anxiety was 5.5% in the first group (≤4 days), accompanied by a mean HADS score (± SD) of 2.2 (± 2.5). In the second group (5-9 days), the prevalence of anxiety was 18.1%, along with a mean HADS score (± SD) of 2.8 (± 2.9). The third group (≥10 days) reported the highest percentage of anxiety prevalence at 36.3% and had a mean HADS score (± SD) of 4.6 (± 3.3). The prevalence of depression was 9.9% in the first group (≤4 days), accompanied by a mean HADS score (± SD) of 1.7 (± 2.3). In the second group (5-9 days), the prevalence of depression was 10.6%, along with a mean HADS score (± SD) of 2.1 (± 2.6). The third group (≥10 days) reported the highest percentage of depression prevalence at 16.9% and had a mean HADS score (± SD) of 3.5 (± 2.9) (**Table 1**).

### The association between accompanying days and depression/anxiety among family caregivers

**Figure 2:**
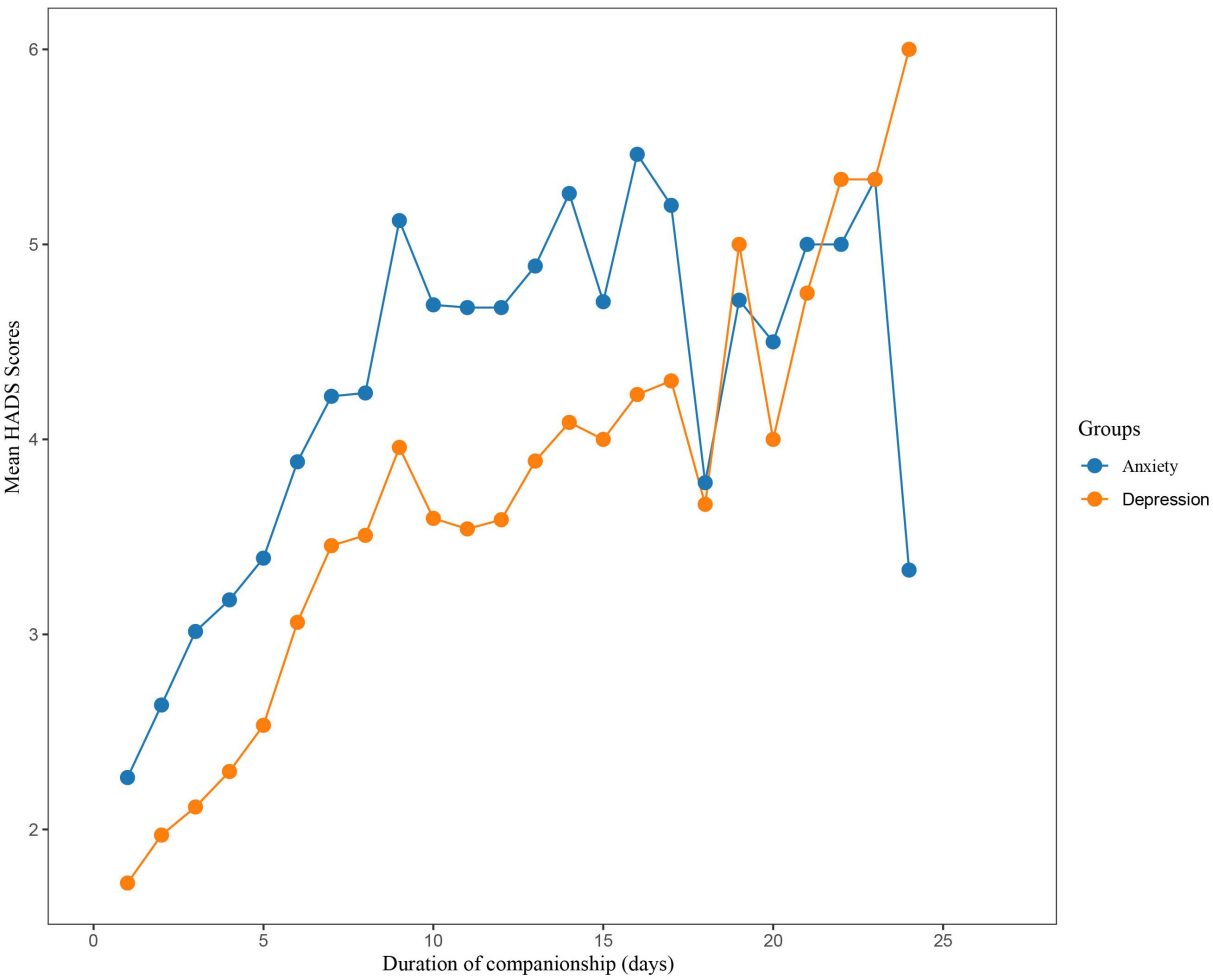
Family caregivers’ HADS scores alongside accompanying days.

**Figure 3:**
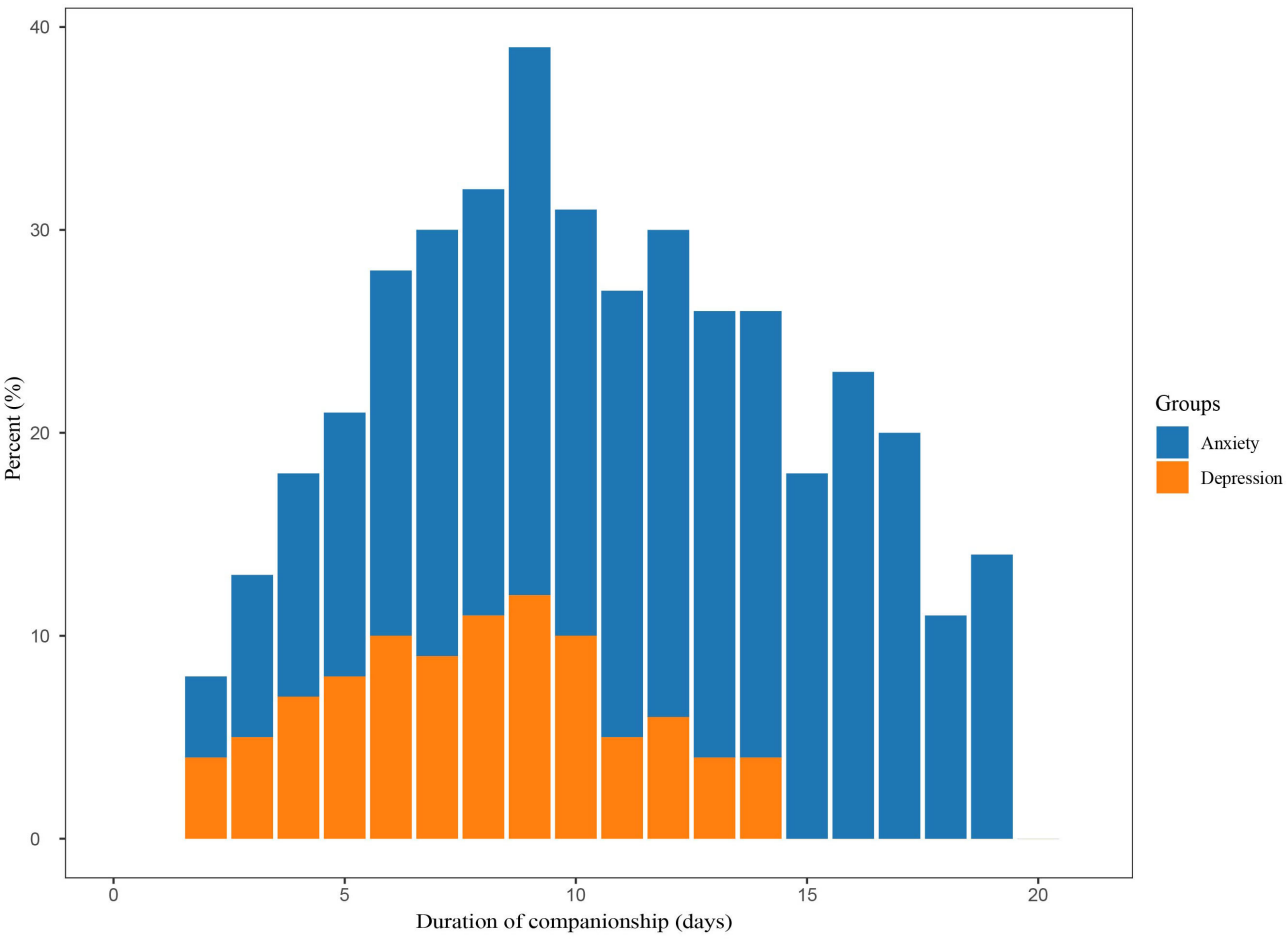
Anxiety and depression were defined using a cut-off score of 8 or higher base on their respective subscales of the HADS. HADS, Hospital Anxiety and Depression Scale.

**Figure 2** displays a gradual increase in anxiety and depression scores among family carers as the duration of concomitant care increased. The highest β value was observed in Group 1 (until 4 days accompaniment), suggesting that the risk of anxiety and depression increased most rapidly during the first 4 days of concomitant care. Specifically, the anxiety scores showed a steeper upward trend compared to depression scores, with the highest recorded anxiety score of 5.4 observed around the 15th day of concomitant care. Depression scores also showed an upward trend with prolonged concomitant care, yet no significant decline was noted. Anxiety and depression were diagnosed using a score of ≥8 on the respective HADS subscales. On admission day, none of the family members reported symptoms of anxiety or depression. However, over time, the proportion of family members presenting with suspected anxiety and depression increased significantly, peaking on the 9th day of hospitalization at 38% for anxiety and 12% for depression. The subsequent proportional incidence of anxiety and depressive symptoms among family members gradually declined, with depression symptoms reaching 0% by day 14 (as illustrated in **Figure 3**) while anxiety symptoms remained steady at 12%.

### Mixed linear regression analysis

**Table 2** demonstrates significantly higher anxiety scores among family caregivers within the first group (≤4 days), with Model I indicating a β value of 0.27, 95% CI (0.16 to 0.39), *p*-value <0.001; Model II indicating a β value of 0.27, 95% CI (0.16 to 0.39), *p*-value <0.001; and Model III indicating a β value of 0.27, 95% CI (0.16 to 0.39), *p*-value <0.001. For family caregivers within the second group (5-9 days), the β values from Model I, II, and III were -0.06, 95% CI (-0.21 to 0.09), *p*=0.438, -0.06, 95% CI (-0.21 to 0.09), *p*=0.436, and -0.07, 95% CI (-0.22 to 0.09), *p*=0.399, respectively. In the case of family within the third group (≥10 days), the βvalues for Model I, II, and III were observed as -0.07, 95% CI (-0.18 to 0.04), *p*=0.224, -0.08, 95% CI (-0.19 to 0.04), *p*=0.221, and -0.15, 95% CI (-0.29 to -0.01), *p*=0.059, respectively.

**Table 2:** The relationship between accompanying days and risk of anxiety and depression.

Days(Events ^a^/No. ^b^)	Model I		Model II		Model III	
	β (95% CI)	*p*-value	β (95% CI)	*p*-value	β (95% CI)	*p*-value
Anxious						
≤4 (34/204)	0.27, (0.16~0.39)	<0.001	0.27, (0.16 ~0.39)	<0.001	0.27, (0.16 ~0.39)	<0.001
5~9 (24/131)	-0.06, (-0.21~0.09)	0.438	-0.06, (-0.21 ~0.09)	0.436	-0.07, (-0.22 ~0.09)	0.399
≥10 (15/42)	-0.07, (-0.18~0.04)	0.224	-0.08, (-0.19 ~0.04)	0.221	-0.15, (-0.29 ~-0.01)	0.059
Depression						
≤4 (23/204)	0.19, (0.10~0.29)	<0.001	0.19, (0.10 ~0.29)	<0.001	0.19, (0.09 ~0.28)	<0.001
5~9 (16/131)	0.05, (-0.09~0.19)	0.481	0.05, (-0.09 ~0.19)	0.488	0.06, (-0.08 ~0.20)	0.424
≥10 (7/42)	-0.03, (-0.09~0.03)	0.343	-0.02, (-0.08 ~0.04)	0.479	-0.02, (-0.08 ~0.04)	0.556

Model I: non-adjusted model; Model II: adjusted for age and gender; Model III: adjusted for all variables.

**^a^** The number of anxiety and depression events occurred within each group.

**^b^** Number of family caregivers included all family caregivers within the specific day range for each group.

The risk of depression was found to be significantly higher within the first group (≤4 days) for family caregivers, with Models I, II, and III all indicating a β value of 0.19, 95% CI (0.10 to 0.29), *p*-value < 0.001. Additionally, for home companionship within the second group (5-9 days), Model I suggested a β value of 0.05, 95% CI (-0.09 to 0.19), while both Model II and III indicated a β value of 0.05, 95% CI (-0.09 to 0.19) and -0.06, 95% CI (-0.08 to 0.20), all *p*-value > 0.05.

### Sensitivity analysis

**Figure 4:**
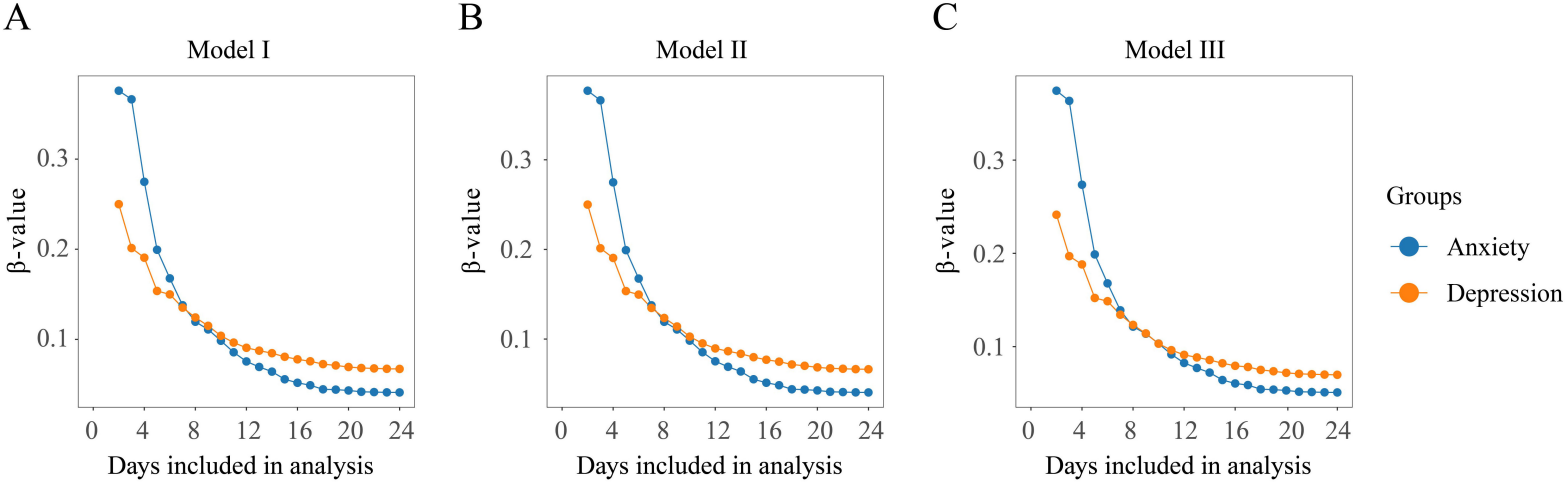
Sensitivity analysis by gradually increasing the number of accompanying days included in the study. **(A)** Model I: non-adjusted model; **(B)** Model II: adjusted for age and gender; **(C)** Model III: adjusted for all variables.

The risk of anxiety and depression among family caregivers reduced gradually with the inclusion of more days of companionship in the study. As shown in, the highest risk of anxiety (β = 0.38, 95% CI: 0.18-0.57) and depression (β = 0.25, 95% CI: 0.05-0.45) was observed at the beginning of patients’ admission. The beta values for both anxiety and depression declined as the number of concomitant days increased, with significant decreases observed until day 9, followed by a levelling off. From day 19, the beta values remained essentially fixed at 0.04 (95% CI, -0.01 to 0.10) for anxiety and 0.07 (95% CI, 0.02-0.12) for depression (**Figure 4**).

## Discussion

During treatment, patients may be susceptible to anxiety and depression, and their accompanying family members, who serve as primary sources of support and stress, may also experience anxiety and depression (30). Our study identified several factors that contribute to anxiety and depression among family caregivers, including the gender of the family caregivers, the relationship with the patient, and the family caregivers’ health status (presence of hypertension, diabetes, coronary heart disease, hyperlipidemia, and stroke). Additionally, the patient’s treatment outcome, the presence of surgery, and the availability of health insurance were also found to influence the anxiety and depression of the family caregivers (**Supplementary Table S1**). These findings are consistent with previous research (31).

The theory of psychological stress posits that the strength of the stress response is linked to the strength of the stressor. For family caregivers, the transition into their new role and the exposure to the unfamiliar hospital environment and surgical procedures can serve as potent stressors. These factors may trigger intense feelings of anxiety and other profound psychological stress reactions that manifest after they begin accompanying the patient following admission. This not only negatively impacts the mental and physical health of the family caregivers but also reduces the quality of care and increases the psychological burden on the patient, ultimately reducing the overall quality of treatment (32).

In our study, we found a high prevalence of anxiety and depression among family caregivers. The HADS questionnaire revealed that 23.5% of family caregivers experienced anxiety symptoms, while 13.1% exhibited symptoms of depression. The prevalence of anxiety in our study population is significantly higher than that of the general population, which is estimated to be around 7.3% according to a global systematic review and meta-regression (33). Our findings also surpass the prevalence of anxiety symptoms reported in a meta-analysis focusing on family caregivers of cancer patients, which stood at 8.9% (34). The proportion of companion anxiety increased as the length of hospitalization with family caregivers became longer (35).

Interestingly, our results are more comparable to a study conducted during the COVID-19 pandemic, which found that approximately 19.52% of family caregivers of patients with COVID-19 experienced anxiety symptoms (36). This similarity suggests that the pandemic context had a significant influence on caregiver anxiety levels. The higher prevalence of anxiety symptoms in our study can be attributed to several factors related to the pandemic situation, including caring for COVID-19 positive patients, strict limitations on caregiver changes, the stressors associated with a surgical setting, and the overall uncertainty and isolation experienced during the pandemic period.

Our findings underscore the substantial mental health impact of caregiving during the COVID-19 pandemic, particularly in a surgical setting. This highlights the pressing need for targeted support and interventions to address the psychological well-being of family caregivers facing such challenging circumstances. Recognizing the heightened vulnerability of family caregivers during this time is crucial in developing effective strategies to mitigate the adverse effects of caregiving on mental health and to provide the necessary resources and support to help family caregivers cope with the unique challenges posed by the pandemic.

The mean anxiety score for all family caregivers was 3.4, while the mean depression score was 2.6. The study findings indicate a relatively lower incidence of anxiety and depression compared to the rates reported by Sallim AB et al. (anxiety: 43.6%, depression: 34.0%). This difference may be attributed to variations in the study populations and the duration of companionship. Specifically, the previous study concentrated on family companionship for Alzheimer’s Disease patients, which necessitates prolonged periods of time and greater emphasis on companion care (37). Our study included patients with various diseases, such as cancer, cardiovascular diseases, and other conditions. The sample sizes differed among the disease groups, which may have influenced the results. For instance, family caregivers of cancer patients may experience higher levels of psychological distress due to the complexity and uncertainty associated with the disease (38). Moreover, the course and prognosis of different diseases can have varying impacts on the anxiety and depression levels of family caregivers (12). However, we employed different models that accounted for the specific diseases, our results remained stable, indicating that the observed associations between hospital accompaniment duration and family caregivers’ anxiety and depression levels were consistent across various disease groups.

Proctor R et al. reported a risk of correlation (β) between patient companionship days and anxiety of 0.26 in their study of dementia patient family companions. However, their study was limited to linear regression analysis and did not use further treatments such as linear mixed effects models for analysis or grouping of days (39).A meta-analysis revealed that the length of companionship was not associated with family companionship anxiety and depression. However, only eight of the studies included in the meta-analysis provided data on the relationship between the length of companionship and family companionship anxiety and depression. Moreover, the total duration of companionship across studies varied widely (10 days to 7.1 years) (40).In our cohort study, we grouped family caregivers by the number of days of companionship and observed a non-linear positive correlation between the duration of family caregivers’ companionship and their anxiety levels. Accounting for covariates such as age, gender, race and ethnicity, education level, household income, marital status, diabetes, metabolic syndrome, smoking, and drinking status, we found that the risk of anxiety and depression was significantly higher for patient companionship in the first group (≤4 days) (β, 0.27; 95% CI, 0.16 to 0.39), which aligns with previous findings. For the second group (5-9 days), family companionship anxiety and depression showed a more stable period, with β values of -0.07 (95% CI, -0.22 to 0.09) for anxiety and 0.06 (95% CI, -0.08 to 0.20) for depression. For the third group (≥10 days), both anxiety and depression showed a noticeable decrease over time, with β values of -0.07 (95% CI, -0.18~0.04) for anxiety and -0.03(95% CI, -0.09~0.03) for depression, which may be attributed to the stabilization of the patient’s condition (e.g., clear diagnosis or completed surgery), increased familiarity with the hospital environment and health care staff by the family, and hospital education interventions.

We conducted a sensitivity analysis due to variations in the number of days family members spent with the patient. We observed a very low percentage of individuals spent more than 15 days with the family. Our analysis revealed that the β values for repeated measures of anxiety and depression, compared to single measures for other Variables, were only minimally impacted when included in the multivariate mixed effects linear model. It is important to note that this observation was consistent across all three models included in our analysis. Our study aimed to evaluate the relationship between the duration of companionship and family caregivers’ risk of developing anxiety and depression. To this end, we incrementally increased the number of companion days to assess changes in risk. Results were consistent with previous analyses using grouped linear mixed-effects models, showing that the risk of anxiety and depression decreased as the length of family companionship increased. Further analyses, which included treatment with continuous and categorical variables, confirmed the stability of these results for anxiety and depression. As reported in previous studies, family caregivers often seek advice from medical staff to manage psychological distress. Our sensitivity analysis showed that family caregivers during hospital admission had a higher risk of anxiety and depression. Thus, we recommend offering psychological guidance at the time of admission to improve family caregivers’ comprehension of necessary psychological support and routine care (41). Based on our analysis, anxiety and depression were found to peak at approximately day 9. As a result, we suggest limiting family companionship to no more than 9 days and considering changing family caregivers at around 4 days to mitigate the risk of negative psychological outcomes. If it is not feasible to change family caregivers, healthcare professionals should monitor the emotional state of both patients and their family caregivers closely and provide timely psychological guidance and communication to reduce the risk of anxiety and depression. If necessary, family caregivers should be advised to seek counseling and treatment from psychological specialists.

Our study has several limitations that should be considered when interpreting the results. First, this study was conducted during the COVID-19 pandemic, which introduces unique contextual factors that may have influenced our results. The pandemic-related mental strain could have significantly impacted the anxiety and depression levels observed in our study population. Second, our study was conducted in a single center in China, which may limit the generalizability of our findings to other healthcare settings or cultural contexts. Third, we used self-reported questionnaires to assess anxiety and depression, which may be subject to response bias.

Our study involved multiple statistical tests, which may increase the risk of Type I errors. Some of our findings, particularly those with *p-*values close to the significance threshold, should be interpreted with caution and viewed as exploratory rather than definitive. Future studies should aim to replicate these results in different populations and settings to confirm the robustness of the associations we observed.

Our study findings should be interpreted within the specific context of Chinese culture and the COVID-19 pandemic. The Chinese cultural emphasis on family responsibility may place greater pressure on family caregivers, while the cultural tendency to hide emotional distress may lead to an underestimation of their anxiety and depression levels.

Additionally, due to the single-center design, data collection was limited to general surgery patients with a single disease type, potentially limiting the generalizability of our findings. Potential residual confounding factors, such as patient disease type, condition, and prognosis, cannot be fully excluded. Lastly, the lack of a standardized definition of family companionship may have impacted our results.

## Conclusion

This study found that during the COVID-19 pandemic, 23.5% of family caregivers of general surgery patients experienced anxiety symptoms, and 13.1% experienced depression symptoms. We observed differences in anxiety and depression risks across three groups of caregivers based on accompanying days (≤4 days, 5-9 days, and ≥10 days). The first group (≤4 days) showed significantly higher risks, suggesting that the initial caregiving period may be particularly challenging for mental health.

## Data Availability

The raw data supporting the conclusions of this article will be made available by the authors, without undue reservation.
