# Supplementary material for: Association between accompanying duration and anxiety/depression among family caregivers: a prospective cohort study in China during the COVID-19 pandemic
Source: Front Psychiatry. 2024 Nov 8;15:1411500. doi: 10.3389/fpsyt.2024.1411500 (PMC11582043; doi:10.3389/fpsyt.2024.1411500)
Supplement: Supplementary file 1 [file Table1.docx]

Supplement Table 1. Baseline characteristics of family caregivers stratified by Anxious or Depression.

| Variables | **Anxious or Depression** | | |  | **Anxious** | |  | **Depression** | |  |
| --- | --- | --- | --- | --- | --- | --- | --- | --- | --- | --- |
|  | Total (n = 207) | No (n = 165) | Yes(n = 42) | *p* | No (n = 173) | Yes(n = 34) | *p* | No(n = 184) | Yes(n = 23) | *p* |
| **Age, Median (IQR)** | 55.2 ± 13.7 | 55.7 ± 13.3 | 53.1 ± 15.3 | 0.273 | 55.3 ± 13.5 | 54.9 ± 14.9 | 0.873 | 55.9 ± 13.4 | 49.8 ± 15.4 | 0.044 |
| **Sex, n (%)** |  |  |  | 0.562 |  |  | 0.894 |  |  | 0.325 |
| Male | 71 (34.3) | 55 (33.3) | 16 (38.1) |  | 59 (34.1) | 12 (35.3) |  | 61 (33.2) | 10 (43.5) |  |
| Female | 136 (65.7) | 110 (66.7) | 26 (61.9) |  | 114 (65.9) | 22 (64.7) |  | 123 (66.8) | 13 (56.5) |  |
| **BMI.comp, Median (IQR)** | 23.7 ± 3.5 | 23.5 ± 3.3 | 24.2 ± 4.3 | 0.277 | 23.7 ± 3.5 | 23.3 ± 3.6 | 0.57 | 23.4 ± 3.3 | 25.3 ± 4.9 | 0.018 |
| **Hospitaltype, n (%)** |  |  |  | 0.852 |  |  | 0.984 |  |  | 0.347 |
| Emergency admission | 91 (44.0) | 72 (43.6) | 19 (45.2) |  | 76 (43.9) | 15 (44.1) |  | 83 (45.1) | 8 (34.8) |  |
| Non-emergency admission | 116 (56.0) | 93 (56.4) | 23 (54.8) |  | 97 (56.1) | 19 (55.9) |  | 101 (54.9) | 15 (65.2) |  |
| **Days, Mean ± SD** | 7.0 ± 4.7 | 6.3 ± 4.1 | 9.8 ± 5.7 | < 0.001 | 6.5 ± 4.5 | 9.9 ± 4.9 | < 0.001 | 6.9 ± 4.5 | 8.3 ± 5.7 | 0.152 |
| **Work, n (%)** |  |  |  | 0.042 |  |  | 0.078 |  |  | 0.027 |
| Employed | 91 (44.0) | 78 (47.3) | 13 (31) |  | 81 (46.8) | 10 (29.4) |  | 85 (46.2) | 6 (26.1) |  |
| Unemployed | 70 (33.8) | 56 (33.9) | 14 (33.3) |  | 58 (33.5) | 12 (35.3) |  | 63 (34.2) | 7 (30.4) |  |
| Other | 46 (22.2) | 31 (18.8) | 15 (35.7) |  | 34 (19.7) | 12 (35.3) |  | 36 (19.6) | 10 (43.5) |  |
| **Race, n (%)** |  |  |  | 0.365 |  |  | 0.302 |  |  | 0.21 |
| Han | 205 (99.0) | 164 (99.4) | 41 (97.6) |  | 172 (99.4) | 33 (97.1) |  | 183 (99.5) | 22 (95.7) |  |
| Other | 2 ( 1.0) | 1 (0.6) | 1 (2.4) |  | 1 (0.6) | 1 (2.9) |  | 1 (0.5) | 1 (4.3) |  |
| **Education, n (%)** |  |  |  | 0.349 |  |  | 0.566 |  |  | 0.64 |
| Primary school and below | 98 (47.3) | 81 (49.1) | 17 (40.5) |  | 83 (48) | 15 (44.1) |  | 89 (48.4) | 9 (39.1) |  |
| Secondary school | 88 (42.5) | 66 (40) | 22 (52.4) |  | 71 (41) | 17 (50) |  | 76 (41.3) | 12 (52.2) |  |
| College or above | 21 (10.1) | 18 (10.9) | 3 (7.1) |  | 19 (11) | 2 (5.9) |  | 19 (10.3) | 2 (8.7) |  |
| **Maritalstatus, n (%)** |  |  |  | 0.101 |  |  | 0.307 |  |  | 0.021 |
| Single | 16 ( 7.7) | 10 (6.1) | 6 (14.3) |  | 12 (6.9) | 4 (11.8) |  | 11 (6) | 5 (21.7) |  |
| Married | 191 (92.3) | 155 (93.9) | 36 (85.7) |  | 161 (93.1) | 30 (88.2) |  | 173 (94) | 18 (78.3) |  |
| **Smoke, n (%)** | 43 (20.8) | 36 (21.8) | 7 (16.7) | 0.463 | 37 (21.4) | 6 (17.6) | 0.623 | 40 (21.7) | 3 (13) | 0.423 |
| **Drink, n (%)** | 34 (16.4) | 25 (15.2) | 9 (21.4) | 0.327 | 26 (15) | 8 (23.5) | 0.221 | 30 (16.3) | 4 (17.4) | 1 |
| **Religion, n (%)** | 117 (56.5) | 95 (57.6) | 22 (52.4) | 0.544 | 98 (56.6) | 19 (55.9) | 0.934 | 105 (57.1) | 12 (52.2) | 0.655 |
| **Economy, n (%)** |  |  |  | 0.071 |  |  | 0.022 |  |  | 0.091 |
| No economic difficulties | 180 (87.0) | 147 (89.1) | 33 (78.6) |  | 155 (89.6) | 25 (73.5) |  | 163 (88.6) | 17 (73.9) |  |
| Economic difficulties | 27 (13.0) | 18 (10.9) | 9 (21.4) |  | 18 (10.4) | 9 (26.5) |  | 21 (11.4) | 6 (26.1) |  |
| **Hypothesis, n (%)** | 38 (18.4) | 28 (17) | 10 (23.8) | 0.307 | 29 (16.8) | 9 (26.5) | 0.181 | 34 (18.5) | 4 (17.4) | 1 |
| **Diabetes, n (%)** | 14 ( 6.8) | 13 (7.9) | 1 (2.4) | 0.309 | 13 (7.5) | 1 (2.9) | 0.474 | 14 (7.6) | 0 (0) | 0.374 |
| **First nursing, n (%)** | 80 (38.6) | 62 (37.6) | 18 (42.9) | 0.53 | 67 (38.7) | 13 (38.2) | 0.957 | 69 (37.5) | 11 (47.8) | 0.338 |
| **Relation, n (%)** |  |  |  | 0.723 |  |  | 0.995 |  |  | 0.648 |
| Spouse | 112 (54.1) | 89 (53.9) | 23 (54.8) |  | 94 (54.3) | 18 (52.9) |  | 102 (55.4) | 10 (43.5) |  |
| Daughter/son | 61 (29.5) | 46 (27.9) | 15 (35.7) |  | 50 (28.9) | 11 (32.4) |  | 51 (27.7) | 10 (43.5) |  |
| Parents | 19 ( 9.2) | 17 (10.3) | 2 (4.8) |  | 16 (9.2) | 3 (8.8) |  | 17 (9.2) | 2 (8.7) |  |
| [Brothers/Sisters](" \l "\\javascript:;" \o "file:///C:\\Users\\jiapeng\\AppData\\Local\\youdao\\dict\\Application\\9.1.6.0\\resultui\\html\\index.html#\\javascript:;) | 4 ( 1.9) | 4 (2.4) | 0 (0) |  | 4 (2.3) | 0 (0) |  | 4 (2.2) | 0 (0) |  |
| Other | 11 ( 5.3) | 9 (5.5) | 2 (4.8) |  | 9 (5.2) | 2 (5.9) |  | 10 (5.4) | 1 (4.3) |  |
| **Only child, n (%)** | 33 (15.9) | 29 (17.6) | 4 (9.5) | 0.203 | 30 (17.3) | 3 (8.8) | 0.215 | 29 (15.8) | 4 (17.4) | 0.768 |
| **Family conflict, n (%)** | 6 ( 2.9) | 5 (3) | 1 (2.4) | 1 | 5 (2.9) | 1 (2.9) | 1 | 5 (2.7) | 1 (4.3) | 0.511 |
| **Childcare, n (%)** | 67 (32.4) | 54 (32.7) | 13 (31) | 0.826 | 57 (32.9) | 10 (29.4) | 0.687 | 59 (32.1) | 8 (34.8) | 0.793 |
| **Living with patients, n (%)** | 153 (73.9) | 121 (73.3) | 32 (76.2) | 0.707 | 128 (74) | 25 (73.5) | 0.956 | 134 (72.8) | 19 (82.6) | 0.314 |
| **Patient sex, n (%)** |  |  |  | 0.218 |  |  | 0.644 |  |  | 0.164 |
| Male | 135 (65.2) | 111 (67.3) | 24 (57.1) |  | 114 (65.9) | 21 (61.8) |  | 123 (66.8) | 12 (52.2) |  |
| Female | 72 (34.8) | 54 (32.7) | 18 (42.9) |  | 59 (34.1) | 13 (38.2) |  | 61 (33.2) | 11 (47.8) |  |
| **Patient age, Median (IQR)** | 60.9 ± 17.7 | 60.6 ± 18.2 | 62.2 ± 15.7 | 0.615 | 60.6 ± 17.6 | 62.4 ± 18.2 | 0.596 | 61.3 ± 17.8 | 57.9 ± 16.3 | 0.385 |
| **Patient insurance, n (%)** |  |  |  | 0.18 |  |  | 0.419 |  |  | 0.266 |
| Urban Residents Basic Health Insurance | 49 (23.7) | 41 (24.8) | 8 (19) |  | 42 (24.3) | 7 (20.6) |  | 44 (23.9) | 5 (21.7) |  |
| New Rural Health Insurance | 135 (65.2) | 109 (66.1) | 26 (61.9) |  | 114 (65.9) | 21 (61.8) |  | 122 (66.3) | 13 (56.5) |  |
| Other | 23 (11.1) | 15 (9.1) | 8 (19) |  | 17 (9.8) | 6 (17.6) |  | 18 (9.8) | 5 (21.7) |  |
| **Patient race, n (%)** |  |  |  | 0.365 |  |  | 0.302 |  |  | 0.21 |
| Han | 205 (99.0) | 164 (99.4) | 41 (97.6) |  | 172 (99.4) | 33 (97.1) |  | 183 (99.5) | 22 (95.7) |  |
| Other | 2 ( 1.0) | 1 (0.6) | 1 (2.4) |  | 1 (0.6) | 1 (2.9) |  | 1 (0.5) | 1 (4.3) |  |
| **Patient work, n (%)** |  |  |  | 0.786 |  |  | 0.151 |  |  | 0.796 |
| Employed | 66 (31.9) | 54 (32.7) | 12 (28.6) |  | 59 (34.1) | 7 (20.6) |  | 60 (32.6) | 6 (26.1) |  |
| Unemployed | 71 (34.3) | 57 (34.5) | 14 (33.3) |  | 60 (34.7) | 11 (32.4) |  | 62 (33.7) | 9 (39.1) |  |
| Other | 70 (33.8) | 54 (32.7) | 16 (38.1) |  | 54 (31.2) | 16 (47.1) |  | 62 (33.7) | 8 (34.8) |  |
| **Patient drink, n (%)** | 51 (24.6) | 40 (24.2) | 11 (26.2) | 0.794 | 43 (24.9) | 8 (23.5) | 0.87 | 47 (25.5) | 4 (17.4) | 0.392 |
| **Patient smoke, n (%)** | 71 (34.3) | 55 (33.3) | 16 (38.1) | 0.562 | 58 (33.5) | 13 (38.2) | 0.597 | 65 (35.3) | 6 (26.1) | 0.379 |
| **Surgery type, n (%)** |  |  |  | 0.33 |  |  | 0.902 |  |  | 0.008 |
| Emergency surgery | 55 (26.6) | 46 (27.9) | 9 (21.4) |  | 47 (27.2) | 8 (23.5) |  | 52 (28.3) | 3 (13) |  |
| Non-emergency surgery | 92 (44.4) | 75 (45.5) | 17 (40.5) |  | 76 (43.9) | 16 (47.1) |  | 85 (46.2) | 7 (30.4) |  |
| Unknown | 60 (29.0) | 44 (26.7) | 16 (38.1) |  | 50 (28.9) | 10 (29.4) |  | 47 (25.5) | 13 (56.5) |  |
| **Cancer, n (%)** | 156 (75.7) | 132 (80) | 24 (58.5) | 0.004 | 137 (79.2) | 19 (57.6) | 0.008 | 142 (77.6) | 14 (60.9) | 0.078 |
| **Surgery, n (%)** | 148 (71.5) | 121 (73.3) | 27 (64.3) | 0.157 | 124 (71.7) | 24 (70.6) | 0.346 | 136 (73.9) | 12 (52.2) | 0.047 |
